# *GPRC6A* Null Mice Exhibit Osteopenia, Feminization and Metabolic Syndrome

**DOI:** 10.1371/journal.pone.0003858

**Published:** 2008-12-03

**Authors:** Min Pi, Ling Chen, Min-Zhao Huang, Wenyu Zhu, Brian Ringhofer, Junming Luo, Lane Christenson, Benyi Li, Jianghong Zhang, P. David Jackson, Pieter Faber, Kurt R. Brunden, John J. Harrington, L. Darryl Quarles

**Affiliations:** 1 The Kidney Institute, University of Kansas Medical Center, Kansas City, Kansas, United States of America; 2 Department of Molecular & Integrative Physiology, University of Kansas Medical Center, Kansas City, Kansas, United States of America; 3 Center for Bone Biology, Clinical Pharmacology, Division/Medicine, Vanderbilt University, Nashville, Tennessee, United States of America; 4 Athersys, Inc., Cleveland, Ohio, United States of America; University of Las Palmas de Gran Canaria, Spain

## Abstract

**Background:**

GPRC6A is a widely expressed orphan G-protein coupled receptor that senses extracellular amino acids, osteocalcin and divalent cations *in vitro*. The physiological functions of GPRC6A are unknown.

**Methods/Principal Findings:**

In this study, we created and characterized the phenotype of *GPRC6A* ^−/−^ mice. We observed complex metabolic abnormalities in *GPRC6A* ^−/−^ mice involving multiple organ systems that express *GPRC6A*, including bone, kidney, testes, and liver. *GPRC6A* ^−/−^ mice exhibited hepatic steatosis, hyperglycemia, glucose intolerance, and insulin resistance. In addition, we observed high expression of *GPRC6A* in Leydig cells in the testis. Ablation of *GPRC6A* resulted in feminization of male *GPRC6A* ^−/−^ mice in association with decreased lean body mass, increased fat mass, increased circulating levels of estradiol, and reduced levels of testosterone. *GPRC6A* was also highly expressed in kidney proximal and distal tubules, and *GPRC6A^−/−^* mice exhibited increments in urine Ca/Cr and PO_4_/Cr ratios as well as low molecular weight proteinuria. Finally, *GPRC6A* ^−/−^ mice exhibited a decrease in bone mineral density (BMD) in association with impaired mineralization of bone.

**Conclusions/Significance:**

*GPRC6A^−/−^* mice have a metabolic syndrome characterized by defective osteoblast-mediated bone mineralization, abnormal renal handling of calcium and phosphorus, fatty liver, glucose intolerance and disordered steroidogenesis. These findings suggest the overall function of GPRC6A may be to coordinate the anabolic responses of multiple tissues through the sensing of extracellular amino acids, osteocalcin and divalent cations.

## Introduction

GPRC6A is a recently identified member of family C of G protein-coupled receptors (GPCRs) that is most closely related to the calcium-sensing receptor CASR [1], [2], [3]. Structural homologies and conservation of specific domains in members of this family of receptors suggest an evolutionary link between extracellular calcium and amino acid-sensing [4], [5], [6]. Indeed, GPRC6A has recently been shown to sense extracellular cations and amino acids and to require both extracellular cations and amino acids for optimal stimulation *in vitro* [3]. This dual sensitivity of GPRC6A to both divalent cations and amino acids is analogous to the related receptor CASR [7]. Compared to CASR, much higher extracellular calcium concentrations are needed to activate GPRC6A [3], [8], and some studies suggest that cations may only be allosteric modulators of GPRC6A [9], whereas others show cation-dependent activation of GPRC6A [3]. The calcimimetic NPS-R578, an allosteric modulator of CASR [10], [11], [12], and osteocalcin, a bone derived calcium binding protein, both enhance the functional responses of GPRC6A to extracellular calcium *in vitro* [3]. The physiologically relevant ligands for and biological function of GPRC6A remain to be determined [13].

GPRC6A is broadly expressed in many tissues and organs, including lung, liver, spleen, heart, kidney, skeletal muscle, testis, brain and bone [1], [2], [3], [14]. The amino acid, osteocalcin, and divalent calcium ligand specificity of this receptor and its wide tissue distribution implicate GPRC6A multiple processes. For example, GPRC6A may be a candidate for the elusive extracellular calcium-sensing mechanism known to be present in osteoblasts [3], [15], [16], [17], [18], which respond to high local Ca^2+^ concentrations (in the range of 8 to 40 mM)[19], amino acids and osteocalcin in the bone microenvironment. GPRC6A is also a candidate for the putative osteocalcin receptor regulating energy metabolism [20]. To determine the function of GPRC6A, we created mice lacking this receptor. We found a complex multiorgan, metabolic-like syndrome in *GPRC6A* ^−/−^ mice that suggests that GPRC6A is involved in nutritional pathways coordinating the metabolic activity of multiple tissues in response to changes in extracellular amino acids and divalent cations.

## Results

### Creation and Gross Phenotype of *GPRC6A* Deficient Mice

**Figure 1:**
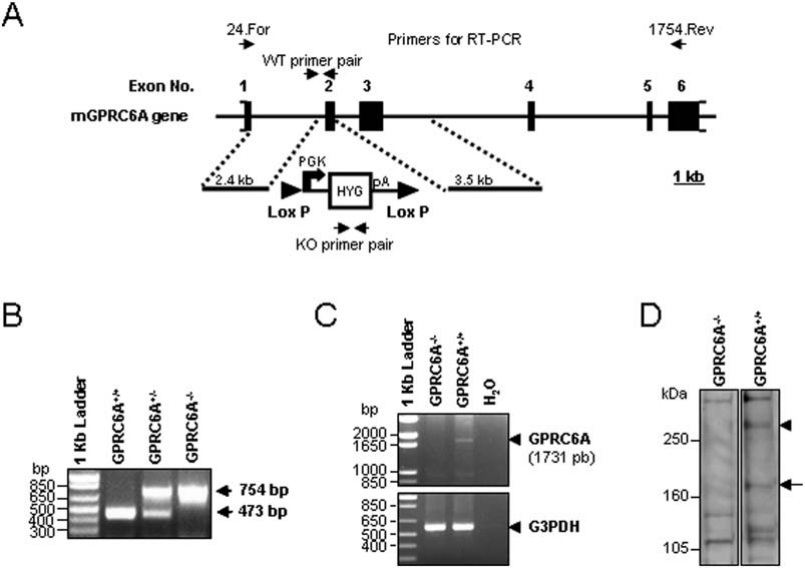
Generation of *GPRC6A* knockout mice. (A) *GPRC6A* knockout targeting strategy. (B) Mouse genotyping by PCR shows the presence of exon 2 in wild-type (*GPRC6A* ^+/+^) and its absence in homozygous *GPRC6A* knockout (*GPRC6A* ^−/−^) mice. RT-PCR analysis (C) and Western Blot analysis (D) of *GPRC6A* expression in kidney from *GPRC6A* ^+/+^ and *GPRC6A* ^−/−^ mice. RT-PCR condition, primer set and anti-mGPRC6A antibody are described in Materials and Methods.

We selectively deleted exon 2 of the mouse *GPRC6A* gene (Fig. 1A). Wild-type *GPRC6A* ^+/+^, heterozygous *GPRC6A* ^+/−^, and homozygous *GPRC6A* ^−/−^ mice were genotyped by PCR (Fig. 1B) and each genotype was found to be born at the expected Mendelian frequencies from heterozygous breeding pairs, however, breeding pairs consisting of *GPRC6A* ^−/−^×*GPRC6A* ^−/−^ had litter sizes that were roughly half that of *GPRC6A* ^−/−^×*GPRC6A^+/+^* or *GPRC6A^+/^* ^−^ and *GPRC6A* ^+/−^ breeding pairs, respectively (Data not shown). In addition, full-length *GPRC6A* transcripts and proteins were documented to be absent from various tissues of *GPRC6A* ^−/−^ mice by RT-PCR (Fig. 1C) and Western Blot (Fig. 1D). *GPRC6A* ^−/−^ mice (as well as heterozygous *GPRC6A* ^+/−^ mice) were similar in gross appearance, body weight and body length to wild-type littermates (data not shown). There were no identified abnormalities in gait or physical activity between wild-type and *GPRC6A* ^−/−^ mice.

### *GPRC6A* Deficiency is Associated with Testicular Feminization and Reduced Testosterone Levels

**Figure 2:**
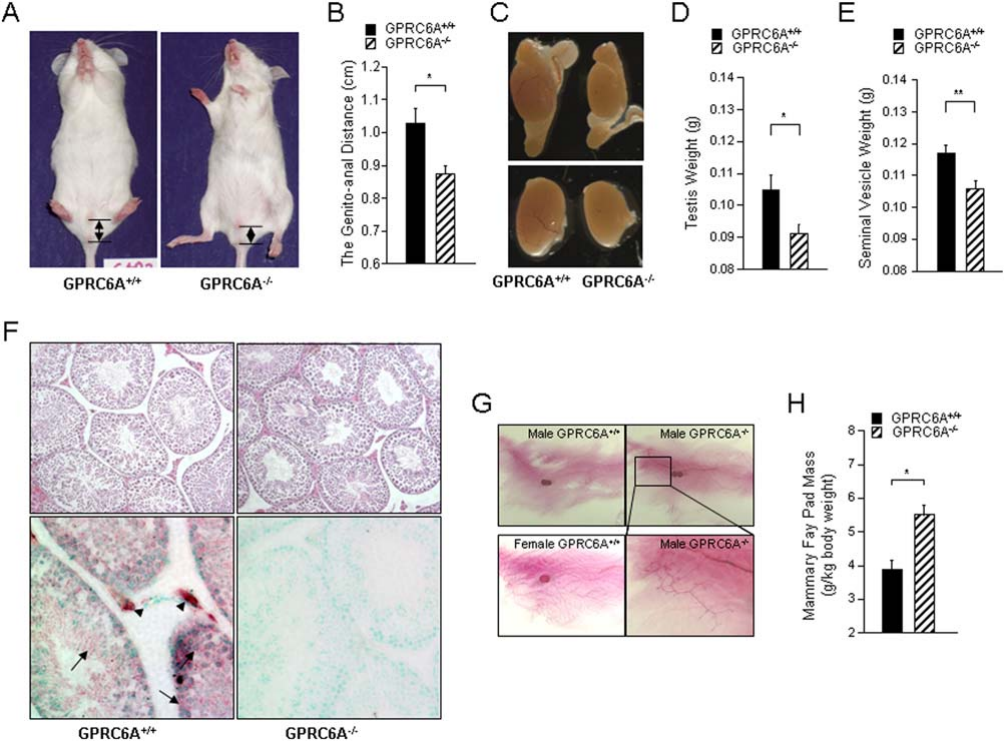
Characterization of reproductive system of male *GPRC6A* ^−/−^ mice. (A) Gross appearance of male *GPRC6A* ^−/−^ mice. The genito-anal distance is demarcated by arrows. (B) Comparison of the genito-anal distance in 16 week-old *GPRC6A^+/+^* and *GPRC6A* ^−/−^ male mice. (C) Gross appearance of testes of male *GPRC6A^+/+^* and *GPRC6A* ^−/−^ at age of 16 week-of-age. Upper panel shows testis and epididymis (magnification 10×) and lower panel shows dissected testis (magnification 20×) viewed under dissecting microscope. (D and E) Comparison of testicular weight (D) and seminal vesicle weight (E) in 16 week-old *GPRC6A^+/+^* and *GPRC6A* ^−/−^ mice. (F) H&E stained sections of testicular histology (upper panel; 200× magnification) and *in-situ* for *GPRC6A* in the testis (lower panel; 400× magnification) from *GPRC6A^+/+^* and *GPRC6A* ^−/−^ mice. The arrow heads depict sites of high *GPRC6A* expression in Leydig cells, and the arrows indicate that *GPRC6A* is also expressed in lower amounts in sertoli cells, spermatogonia and spermatids. (G) Mammary gland abnormalities in male *GPRC6A* ^−/−^ mice at 16 week-old. (H) Comparison of mammary fat pad mass in 16 week-old male *GPRC6A^+/+^* and *GPRC6A* ^−/−^ mice. Data represent the mean±SEM from 6 to 10 mice in each group. * indicates a significant difference from *GPRC6A* ^+/+^ and *GPRC6A* ^−/−^ mice at *p*<0.05, respectively.

On closer inspection, we noted that male *GPRC6A* ^−/−^ mice had feminization of the external genitals (Figs. 2A–C). In 16-week-old male mice, the genito-anal distance (Figs. 2A and B) as well as testicular size (Fig. 2C), weight (Fig. 2D) and the weight of seminal vesicle (Fig. 2E) were significantly reduced in *GPRC6A* ^−/−^ compared to wild-type littermates. No histological abnormality of the testes was noted in *GPRC6A* ^−/−^ mice (Fig. 2F). *GPRC6A* was highly expressed in Leydig cells, and was also expressed in sertoli cells, spermatogonia and spermatids by *in-situ* hybridization analysis (Fig. 2F, lower panel). We also found abnormalities of mammary glands in male *GPRC6A* ^−/−^ mice, as evidence by greater ductal outgrowth in the mammary fat pad (in 10/14 *GPRC6A* ^−/−^ compared to 3/13 mice wild-type male mice) (Fig. 2G) and increased the mammary fat pad mass (Fig. 2H). We found no evidence of embryonic lethality or reduced fertility in homozygous null male or female mice when bred to their respective wild-type mates; however we observed a reduced litter size from breeding pairs consisting of both male and female homozygous null mice.

Next, we compared the serum testosterone and estradiol concentration in 16-week-old mice wild-type and *GPR6CA* ^−/−^ mice. Testosterone concentrations in male *GPRC6A* knockout mice were significantly lower and the estradiol concentrations were significantly higher in male *GPRC6A* ^−/−^ mice compared to wild-type littermates (Table 1). Estradiol levels were not different between wild-type and *GPRC6A* ^−/−^ female mice, although the circulating testosterone levels were lower in female *GPRC6A* null mice (Data not shown).

**Table 1:** Serum and urinary biochemistries from *GPRC6A^+/+^* and *GPRC6A* ^−/−^ mice at age of 16 week old.

		GPRC6A^+/+^	GPRC6A^−/−^
**Serum**	**Calcium (mg/dl)** a	8.23±0.1	8.27±0.19
	**Phosphorus (mg/dl)**	5.18±0.21	6.52±0.18**
	**FGF23 (pg/ml)**	74.51±8.24	89.09±10.71
	**PTH (pg/dl)**	50.6±7.96	50.26±4.91
	**1,25(OH)2 Vit D3 (mg/dl)**	366.17±98.06	251.35±38.56
	**TRAP (U/L)**	4.66±0.55	5.25±0.74
	**Insulin (ng/ml)**	1.78±0.2	0.94±0.15*
	**Glucose (mg/dl)** b	131.67±0.33	168.33±7.33*
	**Cholesterol (mg/dl)** c	170.51±3.38	181.27±5.27
	**Osteocalcin (ng/ml)** c	70.75±5.35	58.07±5.99
	**Testosterone (ng/ml)** c	1.25±0.42	0.19±0.05*
	**Estradiol (pg/ml)** c	16.4±8.59	57.95±17.17*
	**LH (ng/ml)** c	0.58±0.36	1.52±0.5
	**FSH (ng/ml)** c	37.3±2.24	42.84±6.3
**Urine**	**Dpd/Creatinine (mg/mg)**	10.07±1.29	10.73±1.39
	**Calcium/Creatinine Ratio (mg/mg)**	0.13±0.01	0.19±0.02*
	**Phosphorus/Creatinine Ratio (mg/mg)**	3.93±0.28	5.32±0.31**
	**Protein/Creatinine Ratio (mg/mg)**	15.32±1.83	22.65±2.37*

a Serum calcium were measured at 8 week-old mice. Data are mean±SEM from more than 10 individual mice in each group.

b Glucose was measured total blood after over night fasting from more than four mice of *GPRC6A^+/+^* and *GPRC6A* ^−*/*−^ male mice, respectively.

c Serum osterocalcin, testosterone, estradiol, LH and FSH were measured from more than six mice of *GPRC6A^+/+^* and *GPRC6A* ^−*/*−^ male mice, respectively.

* and ^**^ Significant difference from *GPRC6A^+/+^* and *GPRC6A* ^−*/*−^ mice at *p* < 0.05 and *p* < 0.01 respectively.

**Figure 3:**
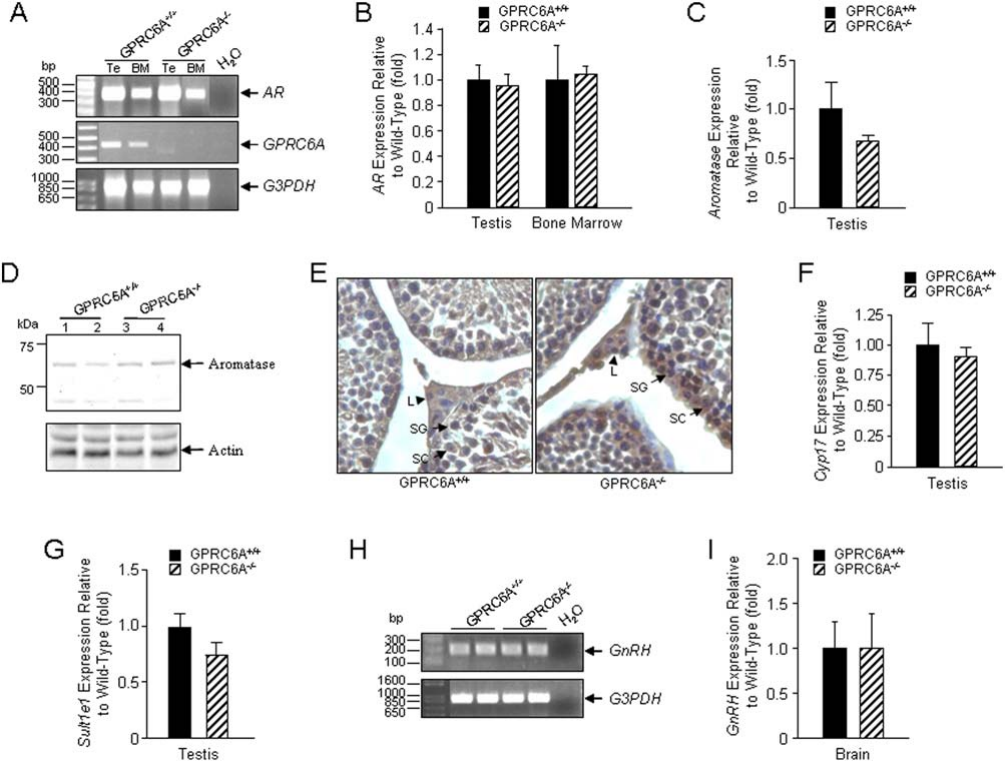
Possible mechanisms underlying altered testosterone/estrogen ratio in *GPRC6A* ^−/−^ male mice. (A and B) RT-PCR and real-time RT-PCR analysis of *androgen receptor* (*AR*) expression. *AR* expression in testis (Te) and bone marrow (BM) was not different between *GPRC6A^+/+^* and *GPRC6A* ^−/−^ male mice. (C) Real time RT-PCR analysis of *aromatase* expression in testis. (D and E) Comparison of the aromatase protein expression in testis from *GPRC6A^+/+^* and *GPRC6A* ^−/−^ male mice. Small increments in aromatase protein expression were observed in *GPRC6A* ^−/−^ male mice by Western blot analysis (D) that was localized by immunhistochemistry (E) to Leydig cells (L, arrow head) and to a lesser degree in sertoli cells (SC), and spermatogonia (SG) (respectively indicated by arrows). (F and G) Real time RT-PCR analysis of *Cyp17* and *Sult1e1* expression in testis. (H and I) RT-PCR and real-time RT-PCR analysis of *GnRH* expression in brain.

Since inactivation of the androgen receptor is reported to lower testosterone levels in mice [21], [22], [23], we examined if loss of *GPRC6A* lowered androgen receptor expression. We observed no difference in the amount of *AR* transcripts in the testis and bone marrow by RT-PCR (Fig. 3A). Real-time RT-PCR also failed to demonstrate any difference in AR expression between *GPRC6A* null and wild-type mice (Fig. 3B). To explore the possibility that the reduction in testosterone was due to increased aromatase-mediated conversion for testosterone to estrogen, we examined aromatase gene (*Cyp19a1*) expression by real-time RT-PCR. The quantitative real-time PCR using primers located in exon 4 and the interface between exons 4 and 5 failed to document any difference in the expression of *Cyp19a1* in *GPRC6A* null mice testis (Fig. 3C). We did, however, detect a small increase in aromatase (CPY19) protein levels in testis of *GPRC6A* ^−/−^ mice by Western Blot analysis (Fig. 3D) that was localized by immunhistochemistry (Fig. 3E) to Leydig cells, sertoli cells, and spermatocytes site where CPY19 is known to be expressed [24]. Aromatase protein level was slightly increased by approximately 11% in testis of *GPRC6A* ^−/−^ male mice (Fig. 3D). The significance of these changes in explaining the alterations in the testosterone and estradiol levels are uncertain, since we did not measure aromatase enzyme activity.

We found no reductions in the expression of P450 17α-hydroxylase gene (*Cyp17*), an enzyme that converts pregnenolone to dehydroandrosterone (DHEA) in the testosterone synthesis pathway [25], or estrogen sulfotransferase gene (*EST/Sult1e1*), which catalyzes the sulfoconjugation and inactivation of estrogens [26], in the *GPRC6A* ^−/−^ male mice testes by real-time PCR (Figs. 3G and H). Similarly, *aromatase* and *Sult1e1* were not different in brain, fat, liver and pituitary of *GPRC6A* deficient and wild-type mice (data not shown). Furthermore, serum follicle-stimulating hormone (FSH) and luteinizing hormone (LH) levels in male mice were not significantly different between wild-type and the *GPRC6A* ^−/−^ male mice (Table 1). Gonadotrophin-releasing hormone gene (*GnRH*) message expression in the brain, however, was not altered in *GPRC6A* null mice (Figs. 3H and I).

### Abnormalities in Renal Function in *GPRC6A* ^−/−^ mice

**Figure 4:**
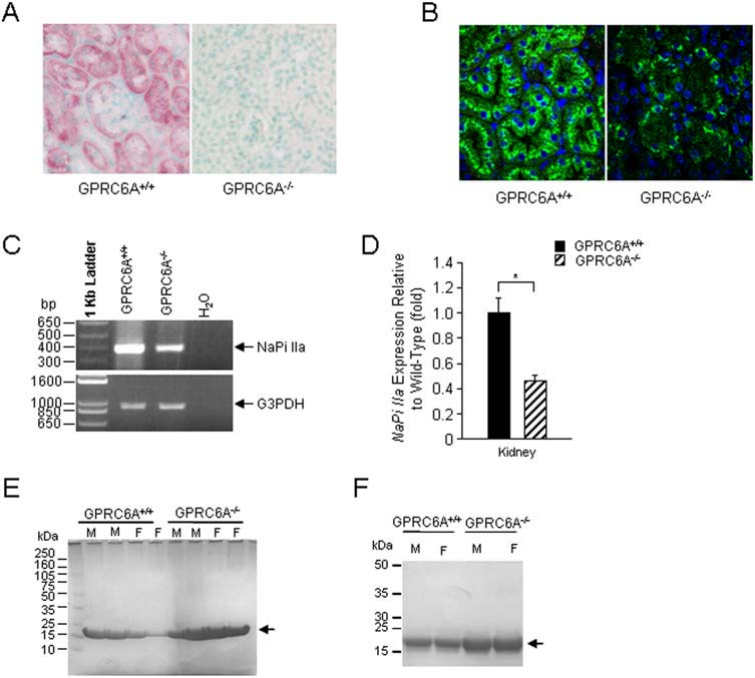
GPRC6A deficiency is associated with a renal phenotype. (A) Expression of *GPRC6A* messenger in kidney by *in-situ* hybridization showing localization of both proximal and distal tubular segments. (B-D) Kidney expression of NaPi IIa. Immunohistochemistry (B) demonstrates decreased Napi IIa protein expression and translocation to the brush border membrane in *GPRC6A* ^−/−^ mice. Loss of *GPRC6A* also resulted in decreased *Napi IIa* message expression by RT-PCR (C) and real-time RT-PCR analysis (D). Data represent the mean±SEM from 6 to 10 mice in each group. * indicates a significant difference from *GPRC6A* ^+/+^ and *GPRC6A* ^−/−^ mice at *p*<0.05, respectively. (E and F) Low molecular weight proteinuria in *GPRC6A* ^−/−^ mice. Western-blot analysis using 4–12% SDS-Page gel and comasis blue staining identified an increase in urinary excretion of a low molecular weight protein in *GPRC6A* ^−/−^ mice (E) that was identified as β2-mcroglobulin by immunobloting with an anti-β2-mcroglobulin antibody (F). The arrow indicates β2-mcroglobulin.

We previously demonstrated that *GPRC6A* is highly expressed in the kidney [3]. We extended these observations by showing that *GPRC6A* is expressed in both proximal and distal tubules by *in situ* hybridization (Fig. 4A). Interestingly, the expression of sodium-phosphate cotransporter, NaPi IIa, (both the protein (Fig. 4B) and transcript (Figs. 4C and D) was decreased ∼50% in *GPRC6A* ^−/−^ mice, suggesting adaptive responses in the kidney to excrete phosphate. We also found that *GPRC6A* ^−/−^ mice had mild but significant increases in urinary calcium and phosphate excretion (calcium/creatinine ratio: 0.19±0.02; phosphorus/creatinine ratio: 5.32±0.31) compared to wild-type controls (calcium/creatinine ratio: 0.13±0.01; phosphorus/creatinine ratio: 3.93±0.28) (Table 1). The mild hypercalciuria was not evident at 6-weeks-of-age, but was present at subsequent ages, whereas the increased urinary phosphate levels were observed only in 16-week old *GPRC6A* ^−/−^ mice. The level of serum phosphorus was also significantly higher in 16 week-old knockout mice (6.52±0.18 mg/dl) compared to wild-type littermates (5.18±0.21 mg/dl) (Table 1). Circulating concentrations of calcium, PTH, FGF23, and 1,25(OH)_2_ vitamin D levels were not significantly different between wild-type and *GPRC6A* ^−/−^ mice (Table 1). In addition, we found that urinary protein excretion was elevated in *GPRC6A* ^−/−^ mice by Western blot analysis (Fig. 4E). Immunohistochemical analysis revealed that this protein band in the urine represents β2-microglobulin (Fig. 4F), providing evidence for abnormalities in the proximal tubule function in *GPRC6A* null mice.

### Metabolic Abnormalities in *GPRC6A* Deficient Mice

**Figure 5:**
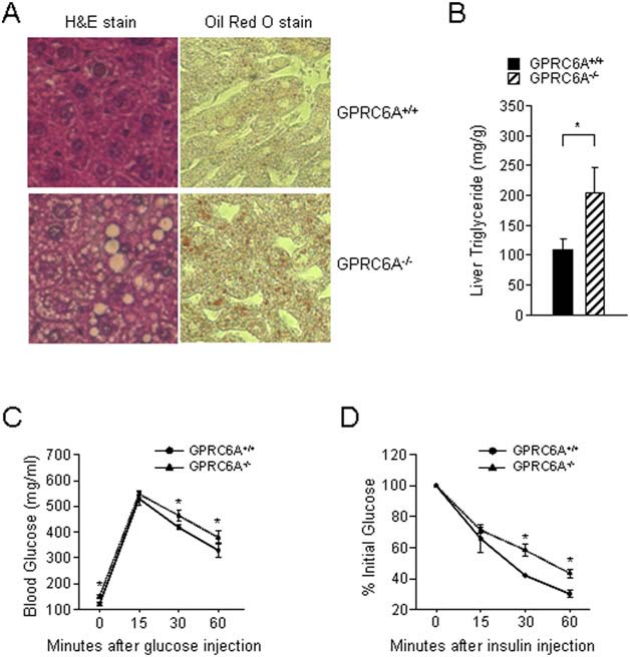
GPRC6A deficiency is associated with a liver phenotype. (A) Histological examination of liver from *GPRC6A* ^−/−^ male mice at age of 16 week old by H & E stained (left panel), Oil Red O stained (right panel). (B) Hepatic triglyceride levels in *GPRC6A* ^+/+^ and *GPRC6A* ^−/−^ male mice at age of 16 week old. Liver triglyceride levels were expressed as mg/g liver tissue. (C and D) GTT (C) and ITT (D) in 3 month-old male *GPRC6A* ^+/+^ and *GPRC6A* ^−/−^ mice. ITT data are presented as percentage of initial blood glucose concentration. Data represent the mean±SEM from more than 5 male mice in each group. * Significant difference from *GPRC6A^+/+^* and *GPRC6A* ^−/−^ mice at *p*<0.05.

We also found that the liver of *GPRC6A* ^−/−^ mice exhibited histological features of hepatic steatosis by H&E and Oil Red O staining (Fig. 5A). Lipid positive droplets were present in hepatocytes of *GPRC6A* ^−/−^ mice but not wild-type mice. This correlated with increased triglyceride content in the livers of GPRC6A^−/−^ mice (Fig 5B). In addition, we found that fasting serum glucose levels were significantly greater and insulin levels were lower in *GPRC6A* ^−/−^ mice compared to wild-type littermates (Table 1). Since fatty liver disease is a manifestation of “metabolic syndrome”, we examined *GPRC6A* ^−/−^ mice for evidence of glucose intolerance. We performed glucose tolerance tests following IP injection of glucose (2 g/kg of body weight) after an overnight fast (GTT), and insulin tolerance tests by IP injection of insulin (0.2 units/kg of body weight) after 6 hours fast (ITT). These tests revealed that *GPRC6A* ^−/−^ mice had a significantly higher serum glucose levels during the GTT and lower sensitivity to insulin than wild-type mice in the ITT (Fig. 5C and D, respectively).

### Musculoskeletal Phenotype of *GPRC6A* Deficient Mice

**Figure 6:**
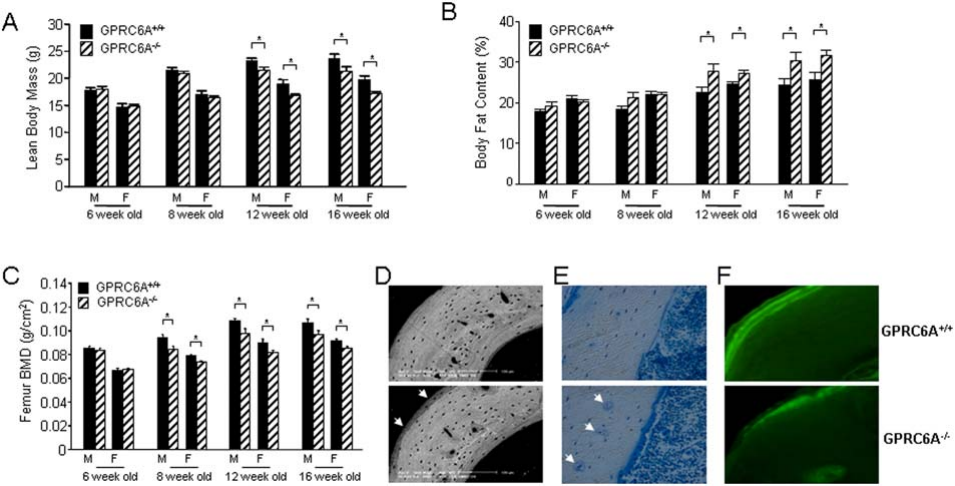
Characterization of the bone phenotype of *GPRC6A* ^−/−^ mice. (A–C) Comparison of the lean body mass (A), fat content (B) and bone mineral density of the femur analysis (C) by PIXImus^TM^ analysis in *GPRC6A* ^+/+^ and *GPRC6A* ^−/−^ mice at ages ranging from 6 to 16 weeks. Data represent the mean±SEM from 6 to 10 mice in each group. * Significant difference from *GPRC6A^+/+^* and *GPRC6A* ^−/−^ mice at *p*<0.05. (D) Backscattered Scanning Electron Microscopy analysis of tibia cortical bone in 16-week-old *GPRC6A^+/+^* (upper panel) and *GPRC6A* ^−/−^ mice (lower panel). The arrows showed the diminished mineralization layer in the bone of *GPRC6A* ^−/−^ mice. (E) Toluidine blue-stained plastic sections of femur from 16-week-old *GPRC6A^+/+^* (upper panel) and *GPRC6A* ^−/−^ mice (lower panel). The arrows showed the unmineralized osteoid surfaces in the bone of *GPRC6A* ^−/−^ mice. (F) Plastic unstained sections of tibia cortical bone viewed under fluorescent light in 16-week-old *GPRC6A^+/+^* (upper panel) and *GPRC6A* ^−/−^ mice (lower panel) prelabeled with twice calcein (double label).

Both male and female *GPRC6A* ^−/−^ mice had a significant reduction in lean body mass compared to wild-type littermates (7.9% and 10% in male and 11.2% and 13% in female *GPRC6A* ^−/−^ mice at 12 and 16 weeks, respectively) as assessed by PIXImus^TM^ densitometry (Fig. 6A). There were no apparent differences, however, in muscle histology between wild-type and *GPRC6A* ^−/−^ mice (data not shown). Body fat as assessed by PIXImus^TM^ densitometry (Fig. 6B) and white fat by gross inspection of various organs, such as testis, were increased in *GPRC6A* ^−/−^ compared to wild-type mice. Both male and female *GPRC6A* ^−/−^ mice did not have the expected age-dependent increase in bone mineral density (BMD). Indeed, BMD was significantly less at 8, 12, and 16 weeks-of-age in *GPRC6A* ^−/−^ mice as compared to age-matched wild type mice (p<0.05) (Fig. 6C). To determine if the decreased bone density might be due to defective mineralization of bone, we performed backscatter EM and bone histological analysis. Backscatter EM of cortical bone demonstrated diminished mineralization surrounding osteocyte lacunae and on the perisoteal and endosteal surfaces (Fig. 6D). Qualitative analysis of bone histology also revealed an increase in unmineralized osteoid surfaces (Fig. 6E) and diffuse calcein labeling of bone compared to the distinct double labels in wild-type mice (Fig. 6F), indicative of impaired mineralization.

## Discussion

Recent data indicate that energy, fat, bone, and glucose metabolism are interrelated through novel hormonal regulatory pathways [20]. We observed a complex phenotype in *GPRC6A* ^−/−^ mice consisting of defective mineralization of bone and impaired osteoblast function in male and female mice, a decrease in lean body mass, an increase in fat mass, hyperphosphatemia and hypercalciuria, hyperglycemia and feminization of male mice associated with altered ratio of estradiol and testosterone, suggesting that *GPRC6A* participates in hormonal control of energy metabolism. Multiple organs and metabolic functions were affected by ablation of *GPRC6A* in mice.

Obesity-dependent metabolic syndrome is associated with impaired glucose tolerance and hepatic steatosis [27]. Similarly, *GPRC6A* ^−/−^ mice had elevated serum glucose levels (Table 1) glucose intolerance, insulin resistance and hepatic steatosis, as evidence by histological presence of fat and biochemical evidence of increase triglyceride content in livers of knockout mice (Fig. 5). The presence of a metabolic-like syndrome in *GPRC6A* ^−/−^ mice suggests that this receptor regulates metabolic pathways involved in glucose and fat metabolism, although these studies do not define the exact target organ of GPCR6A effects or whether this is a direct effect of GPRC6A or an indirect effect related to insulin resistance in *GPRC6A* ^−/−^ mice. Recently, osteocalcin produced by bone has been shown to be a circulating hormone that targets the pancreas to increase insulin secretion and adipocytes to increase adiponectin production [28]. Osteocalcin (Oc) has also been shown to target an unknown Gαi coupled GPCR [29] and can activate GPRC6A in the presence of extracellular calcium [3]. These observations raise the possibility that GPRC6A, might mediate some of the actions of Oc in vivo and provide a molecular pathway linking bone and energy metabolism. Future studies will be needed to determine if this receptor mediates the effects of osteocalcin on energy metabolism and to clarify the mechanisms underlying insulin resistance and hepatic steatosis in *GPRC6A* ^−/−^ mice.

Another phenotype was feminization of male *GPRC6A* ^−/−^ mice. Low testosterone levels acting observed in *GPRC6A* null mice, through classical genomic mechanisms, could explain the feminization of *GPRC6A* null mice. The phenotype of *GPRC6A* null mouse resembles, but is less severe than, the *AR* null and *Tfm* (Testicular feminized) mouse models [21], [23]. *GPRC6A* null mice, however, have inappropriately normal LH and FSH levels and increased estrogen, whereas the disruption of the genomic actions of AR is associated with increased LH and FSH and low estradiol levels [30]. Our studies do not define the mechanism of the altered testosterone/estrogen ratio. The alterations in circulating sex steroid levels in *GPRC6A* null mice might result from direct actions of the receptor to regulate testosterone biosynthesis or conversion to estrogens in the testis or other tissue, and/or to an indirect effect through potential GPRC6A regulation of the hypothalamus or pituitary gland. The observation that *GPRC6A* is expressed in the hypothalamus (GEO accession GDS565), gonadotrope cells of the anterior pituitary (GEO accession GDS2167), sertoli cells (GEO accession GDS222) and testes (GEO accession GDS2098) as well as the slight increase in level of aromatase protein expression in testis support both possibilities [2], [3], [14]. Testosterone replacement in *GPRC6A* ^−/−^ mice, measurement of aromatase activity and steroid biosynthesis, and assessment of GPRC6A function in other tissues will all be needed to establish the biological significance and mechanism of the observed reduction in testosterone levels in these mice.

The predominant effect of *GPRC6A* deficiency in bone was to impair bone mineralization. It is not clear whether these bone abnormalities represent a direct effect of *GPRC6A* loss from osteoblasts or secondary effects due to potential actions of the concomitant sex steroid hormone abnormalities on bone remodeling. But testosterone deficiency typically leads to increased osteoclast-mediated bone resorption [31], which was not observed in these animals. Further studies will be needed to determine the relative contribution of secondary alterations in sex hormones and primary loss of calcium-sensing receptor responses to the observed bone phenotype in *GPRC6A* null mice. Regardless, the expression of *GPRC6A* in bone and osteoblasts and the resulting bone phenotype raises the possibility that GPRC6A is a candidate for the novel osteoblastic calcium-sensing receptor [3], [15]. that is distinct from CASR [15].

With regard to the kidney, we observed that *GPRC6A* is expressed in both proximal and distal tubules and the ablation of this receptor resulted in increased renal excretion of calcium, phosphate, and β2-microglobulin. The effects of GPRC6A loss of function on urinary calcium excretion is opposite to the hypocalcuria caused by inactivating mutations of the related calcium sensing receptor CASR [32]. It is not clear from our studies if these renal abnormalities associated with disruption of GPRC6A function are direct or indirect. We failed to observe in *GRPC6A* null mice evidence of secondary increase PTH or increased bone turnover that would be expected if *GPRC6A* ablation resulted in a renal calcium leak. Theoretically, a primary decrease in bone formation and decreased buffering capacity for calcium, with consequent increased urinary expression of dietary calcium could account for the relationship between osteopenia and hypercalciuria in *GPRC6A* ^−/−^ mice. Alternatively, a primary effect of GPRC6A to regulate gastrointestinal phosphate transport would be another possible explanation for increased serum and urinary phosphate. There are some enigmatic clinical disorders with features similar to the *GPRC6A* ^−/−^ mice that support the possibility of coordinated effects between bone formation and renal conservation of calcium. In this regard, a subset of male patients with idiopathic osteoporosis and nephrolithiasis have the combined features of decreased osteoblast-mediated bone formation and hypercalciuria, without evidence of hypogonadism, secondary hyperparathyroidism, or abnormal vitamin D levels [33] [34]. It will be interesting to determine if gene polymorphisms of GPRC6A are associated with osteopenic and hypercalciuric clinical disorders. The observed reduction of NaPi IIa in *GPRC6A* ^−/−^ mice and the low molecular weight proteinuria is consistent with a primary proximal tubular defect. The increase in serum phosphate in *GPRC6A* ^−/−^ is unexplained.

In summary, we have shown that GPRC6A has multiple functions as evidenced by abnormalities in *GPRC6A* null mice that include alterations in circulating testosterone and estrogen levels and feminization of male mice, defects of bone density and bone cell function and abnormalities in the renal handling of calcium and phosphate, hyperglycemia and liver steatosis. The ligand profile of GPRC6A, which includes extracellular calcium, calcimimetics, amino acids, and osteocalcin [2], [3], [14], [20], along with the complex phenotype of *GPRC6A* null mice suggests that GPRC6A may represent an anabolic receptor that responds to a variety of nutritional and hormonal signals and may serve to coordinate the functions of multiple organs in response to changes of these ligands.

## Materials and Methods

### ***Generation of GPRC6A Knockout Mice***

The *GPRC6A*-deficient mouse model was created by replacing exon 2 of the *GPRC6A* gene with the hygromycin resistance gene (Fig. 1). To generate the targeting construct, the hygromycin resistance gene under the control of the PGK promoter was cloned into the *Sma* I and *Eco* RV sites of pBS-lox, which was produced by cloning the oligonucleotide Lox71: 5′-ctagataccgttcgtatagcatacattatacgaagttatg-3′ into the *Xba* I and *Bam* HI sites and the oligonucleotide Lox 66: 5′-agcttataacttcgtatagcatacattatacgaacggtag-3′ into the *Hin*d III and *Sal* I sites of pBluescript (Stratagene), to produce pBS-lox-PGK-Hyg. A genomic fragment from intron 1 of *GPRC6A* gene, representing the 5′ homologous targeting region, was amplified by PCR using Advantage 2 Taq polymerase (BD Biosciences) and primers PP 232-Avr: 5′-aaacctagggccattcatgaaaaaatgttgtcctcagatgaccatcc-3′, and PP 233-Avr: 5′-aaacctaggctcactcaacccccatgtccttccaactctagctg-3′, digested with *Avr* II, and cloned into the *Xba* I site of pBS-lox-PGK-Hyg to produce pBS-GPRC6A-Intron 1. A genomic fragment from intron 2 of *GPRC6A*, representing the 3′ homologous targeting region, was then amplified by PCR using Advantage 2 Taq polymerase and primers 298: 5′-aaagtcgacctacattggtccatcgattacattagttcttgg-3′ and PP 299: 5′-aaagtcgacgaggccttgaggtcaaactccagaaccccagag-3′, digested with *Sal* I, and cloned into the *Sal* I site of pBS-GPRC6A-Intron I to produce pGPRC6A-KO. The methods for knocking out the *GPRC6A* gene have been described previously in detail [35]. Briefly, the mouse embryonic cell line RW-4, derived from 129X1/SvJ mouse strain[36] and kindly provided by Stephan Teglund at the Karolinska Institute Center for Transgene Technologies, was transfected by electroporation with *Not* I linearized p*GPRC6A*-KO. Hygromycin resistant embryonic stem cell colonies were picked, expanded, and tested by PCR to identify clones in which the PGK-Hygromycin gene had correctly replaced exon 2 of the *GPRC6A* gene. The correctly mutated embryonic stem clones were injected into blastocysts derived from C57BL/6 3.5 days after mating and implanted into B6CBAF1 pseudopregnant females. The resulting male chimeras were bred with female C57BL/6 mice. Homozygous founders were generated by mating the resulting heterozygous mice. The successful targeting of *GPRC6A* in embryonic stem (ES) cells was confirmed by Southern blot analysis of the genomic DNA from ES cell clones. We observed no apparent differences in the founders generated from different ES cell clones. We focused our studies on founder line 17.

Mice were maintained and used in accordance with recommendations as described (National Research Council. 1985) Guide for the Care and Use of Laboratory Animals DHHS Publication NIH 86-23 , Institute on Laboratory Animal Resources, Rockville, MD) and following guidelines established by the University of Kansas Medical Center Institutional Animal Care and Use Committee.

### PCR primers

The following intron-spanning primer sets were used for RT-PCR: mGPRC6A.24.For: ccagaaagatggccctattga; mGPRC6A.1754.Rev: ctccttactggggcccagtggg; mAndR.F578: caacttgcatgtggatgacc and mAndR.R961: cttgagcaggatgtgggattc. mGnRH.For169: agcactggtcctatgggttg and mGnRH.Rev389: gggccagtgcatctacatct. NaPiII.F248: ccacctatgccatctccagt and NaPiII.R635: accatgctgacaatgatgga; mALP.905F: aacccagacacaagcattcc and mALP.1458R: ctgggcctggtagttgttgt, G3PDH.F143: gaccccttcattgacctcaactaca; G3PDH.R1050: ggtcttactccttggaggccatgt for control RNA loading. The following primer sets were used for real-time PCR: *aromatase* forward primer: tgagaacggcatcatatttaacaac and reverse primer: gcccgtcagagctttcataaag; *Cyp17* forward primer: tggaggccactatccgagaa and reverse primer: tgttagccttgtgtgggatgag; and *Sult1e1* forward primer: tcatgcgaaagggaattatagga and reverse primer: tgcttgtagtgctcatcaaatctct.

### RT-PCR and Real-Time RT-PCR

RT-PCR was performed using two-step RNA PCR (Perkin-Elmer) as previously described [3]. Specific intron-spanning primer sets were to amply the specified transcripts. For quantitative real-time RT-PCR assessment of bone markers expression, we isolated and reverse transcribed 2.0 µg total RNA from long bone of 8-week-old mice as previously described [37]. For quantitative real time RT-PCR assessment of *aromatase*, *CYP17* and *Sutl1e1* genes expression we isolated and reverse transcribed total RNA isolated from testis, brain, fat, liver and pituitary of 33 week-old *GPRC6A^+/+^* (n = 5) and *GPRC6A* ^−/−^ mice (n = 5) as previously described [38] using specific primer sets. For expression of *AR, GnRH* and *NaPi IIa*, the oligo primers sequences were obtained from Primer Bank, a public resource for PCR primers (pga.mgh.harvard.edu/primerbank/) [39], and the threshold cycle (Ct) of tested-gene product from the indicated genotype was normalized to the Ct for cyclophilin A as we have previously described [37].

### Cell Culture and Western Blotting

HEK-293 cells were co-transfected with pcDNA3.mGPRC6A or pcDNA3 plasmid as previously described [3]. An anti-peptide antibody was raised in a rabbit against a peptide (AIHEKMLSSDDHPRRPQIQKC) corresponding to a sequence in the extracellular domain of mouse GPRC6A (in exon 1 of mouse *GPRC6A* gene) produced by Abgent (San Diego, CA). For phospho-ERK, the phospho-ERK1/2 levels were determined by immunoblotting using anti-phospho-ERK1/2 mitogen-activated protein kinase antibody (Cell Signaling Technology) [3]. Urinary β2-mcroglobulin was detected by rabbit polyclonal anti-β2-mcroglobulin antibody (Abcam Inc.). For aromatase expression analysis, the rabbit polyclonal anti-aromatase antibody (Abcam Inc.) and goat anti-rabbit IgG HRP secondary antibody (Santa Cruz Biotechnology Inc.) were used. Mouse anti-Actin antibody (Santa Cruz Biotechnology Inc.) was used for control protein loading.

### PIXImus™ Bone Densitometer Analysis and Bone Histology

Bone mineral density (BMD) of whole skeletons and femurs were assessed at 6, 8, 12, and 16 weeks of age using a PIXImus™ bone densitometer (Lunar Corp.) as previously described [32]. Skeletons of mice were performed as reported previously by our laboratory [32].

### Backscattered Scanning Electron Microscopy

Plastic embedded bone from 8 week-old wild-type and *GPRC6A* knockout mice were cut and polished, and mounted on aluminum sockets with bone surface facing above, sputter-coated with gold and palladium, and examined with field emission scanning electron microscopy (Philips XL30, FEI Company) equipped with a backscatter electron imaging system.

### Immunohistochemistry

Wild-type and GPRC6A mouse kidney were routinely processed and embedded in paraffin. The paraffin sections at thickness of 5 µm were prepared and collected on commercially available, positively charged glass slides (Superfrost Plus, Fisher Scientific). The sections were dried on a hot plate to increase adherence to the slides. Representative sections were de-paraffined and re-hydrated through conventional methods. The sections were digested by 10 mg/ml hyaluronidase for 20 min. Nonspecific protein binding was blocked by incubation with 10% normal goat serum. The sections were incubated in polyclonal rabbit against mouse NaPi IIa (1∶500 dilution) or polyclonal goat anti-human aromatase antibody (1∶200 dilution) (CYP19, Santa Cruz Biotechnology, Inc.) at 4°C overnight. The negative control sections were incubated with 0.01 M PBS. Thereafter, the sections were treated sequentially with FITC-conjugated Donkey anti Rabbit IgG secondary antibody (Jackson Labs). The nucleus was stained with ready to use Hoechst (Sigma).

### Whole-Mounts and Carmine Stain of Mammary Glands

Mice mammary fat pads were excised and fixed for a minimum of 2 h in Carnoy's solution (60% ethanol, 30% chloroform, and 10% glacial acetic acid). The fixed glands were washed in 70% ethanol for 15 min and then rinsed in water for 5 min. The mammary glands were stained overnight at 4°C in carmine alum stain (1 g carmine and 2.5 g aluminum potassium sulfate in 500 ml water).

### In Situ Hybridization

For GPRC6A gene expression, the probe was amplified by RT-PCR using following intron spanning primer: mGPRC6A.189F (in Exon I) cgggat ccagacgaccacaaatccag and mGPRC6A.539R (spanned over Exon II and III) ccaagctt gattcataactcacctgtggc. After RT-PCR, the product was subclone into pre-cutted by *Bam*H I and *Hin*d III of pBluescript SK(+). Using T7 promoter will create sense RNA, and T3 promoter will create Anti-sense RNA ribo-probe.

### Serum and Urine Biochemical Measurements

Serum was collected using a retroorbital bleeding technique. For urine samples collection, mice were placed in metabolic cages (Hatteras Instrument), and urine was collected for 24 h. The urine volume was measured before storage at −70°C.

Serum testosterone and estradiol levels were measured by testosterone enzyme immunoassay test kit and estradiol (E2) enzyme immunoassay test kit from BioCheck, Inc. Follicle stimulating hormone (FSH) and luteinizing hormone (LH) were measured by mouse FSH radioimmunoassay and the mouse LH sandwich assay as described by the University of Virginia Center for Research in Reproduction Ligand and Analysis Core (NICHD (SCCPRR) Grant U54-HD28934). Serum and urinary Calcium was measured by the colorimetric cresolphthalein binding method, and phosphorus was measured by the phosphomolybdate–ascorbic acid method [32]. Serum TRAP was assayed with the ELISA-based SBA Sciences mouseTRAP™ assay. Serum PTH and 1, 25 (OH)_2_ vitamin D were measured the kits from Immutopics, Inc. and Immunodiagnostic system, Ltd., respectively. Serum Fgf23 levels were measured by using FGF-23 ELISA kit (Kainos Laboratories Inc.) following the manufacturer's protocol. Creatinine was measured by the colorimetric alkaline picrate method (Sigma kit 555, Sigma-Aldrich). Urinary protein and Dpd were measured by Bio-Rad and Metra Biosystems, Inc., respectively.

### Metabolic Studies

For glucose tolerance test (GTT) glucose (2 g/kg body weight) was injected intraperitoneally (IP) after an overnight fast, and blood glucose was monitored using blood glucose strips and the Accu-Check glucometer (Roche) at indicated times. For insulin tolerance test (ITT) mice were fasted for 6 hr, injected IP with insulin (0.2 U/kg body weight, Lilly Research Laboratories), and blood glucose levels were measured at indicated times as described [20]. ITT data are presented as percentage of initial blood glucose concentration.

### Statistics

We evaluated differences between groups by one-way analysis of variance. All values are expressed as mean±SEM. All computations were performed using the Statgraphic statistical graphics system (STSC Inc.).
